# Effects of Plastic Mulching and Basal Nitrogen Application Depth on Nitrogen Use Efficiency and Yield in Maize

**DOI:** 10.3389/fpls.2018.01446

**Published:** 2018-10-02

**Authors:** Xiukang Wang, Ning Wang, Yingying Xing, Jia Yun, Huihui Zhang

**Affiliations:** Shaanxi Engineering and Technological Research Center for Conversation and Utilization of Regional Biological Resources, College of Life Sciences, Yan’an University, Yan’an, China

**Keywords:** nitrogen uptake, nitrogen loss, traditional broadcast nitrogen fertilizer, topdressing nitrogen fertilizer, root zone

## Abstract

The demand for increased grain production to support population and consumption growth has led to increased interest in field management approaches that incorporate plastic mulching and fertilization management. The purpose of this study was to investigate the effects of plastic mulching and basal nitrogen (N)-fertilizer application depth on N balance estimations, N use efficiency (NUE) and maize yield. The experiment was conducted in 2014 and 2015 with six treatments: no N fertilizer and no mulching (CK), traditional broadcast N fertilizer with mulching (T0), basal N-fertilizer application at a depth of 6 cm with no mulching (T1), basal N-fertilizer application at a depth of 6 cm with plastic mulching (T2), basal N-fertilizer application at a depth of 12 cm with no mulching (T3) and basal N-fertilizer application at a depth of 12 cm with plastic mulching (T4). Mulching and basal N-fertilizer application depth each had significant effects on grain yield, but there were no significant interactions between them. The highest grain yield was observed in the T2 treatment and was 89.1% and 99.8% higher than the grain yield in the CK treatment in 2014 and 2015, respectively. The N uptake in T2 was 21.3% and 25.3% higher than that in the T0 treatment in 2014 and 2015, respectively. Relative to the value in the T0 treatment, the mean N loss over the 2 years was reduced by 34.6% in T2 and by 39.8% in T4. The basal N-fertilizer application depth of 12 cm yielded an obvious increase in NUE, but a high N residual remained below 50 cm after harvest, indicating the higher potential for N losses. In addition, the field application of this type of fertilizer management would require more labor in the absence of the implementation of mechanization. Based on the results, basal N-fertilizer application a depth of 6 cm without plastic mulching is recommended because it significantly increased grain yield and NUE, reduced N loss and requires no investment in plastic film, which are conducive to food security and environmental conservation.

## Introduction

Drylands occupy approximately 40–41% of Earth’s land area and are home to more than 2 billion people (Zhang X. et al., 2017). In dryland farming systems, soil nutrient deficiency and water shortage are two major factors limiting plant growth and crop yield (Mi et al., 2016). In China, most rainfed agriculture is distributed on the Loess Plateau, which covers approximately 25 million hectares (Zhang et al., 2018). The climate is predominantly semi-arid, with high evaporation and low temperature in the annual planting season (Zhang Y. et al., 2017). Maize growth on the Loess Plateau is suffering from climate change inducing seasonal drought and cold springs. Water scarcity on the Loess Plateau is expected to worsen in the future.

Mulching technologies are common and effective practices to improve crop production worldwide, particularly in dryland areas (Mo et al., 2017). Numerous studies have reported that mulching can increase soil water availability in dryland areas, mainly because of its ability to reduce soil evaporation by preventing capillarity (Liu et al., 2017; Zhang P. et al., 2017). Furthermore, mulches can significantly improve topsoil temperature, which benefits seed germination and root growth during the early stages of plant development (Ren et al., 2017). The use of mulch in agriculture provides many other benefits to the soil by adjusting the microbial biomass (Muñoz et al., 2017), increasing nutrient cycling (Kader et al., 2017), reducing soil CO_2_ emission (Dossou-Yovo et al., 2016), maintaining the soil organic carbon balance (Wang et al., 2016), enhancing soil aggregate stability (Wang et al., 2017), promoting soil enzyme activity (Qian et al., 2015) and suppressing weed infestation (Kołodziejczyk, 2015). In general, among mulching approaches, the application of mulch in combination with appropriate nitrogen (N) fertilizer application or drip fertilization has the best performance in terms of promoting agricultural production. Maize is predominantly cultivated in Loess Plateau. Furthermore, although drip fertigation is widely used in vegetable and fruit plant production, an efficient, practicable, cost-effective drip fertigation system is lacking for maize production.

Numerous studies of crops have focused on the N-fertilizer application rate, the proportion of basal and topdressing N-fertilizer and the controlled release of nitrogen fertilizer (Ning et al., 2017; Wasaya et al., 2017). In some cases, N management is combined with mulching technologies, such as mulching with plastic film, mulching only with ridges or alternating mulching with ridges and furrows (Wang et al., 2015; Jia et al., 2018). Ridge–furrow mulching with plastic film has been used to conserve soil water and improve water use efficiency and crop productivity and has been widely adopted on the Loess Plateau (Fan et al., 2017; Zhang F. et al., 2017).

To address water scarcity problems and increase yields, mulching and N fertilization are usually recommended for smallholders on the Loess Plateau. Despite the widespread use of mulching and N fertilization in rainfed agricultural regions of China, few studies have reported the basal N application depths for maize crops in China or other regions worldwide. In particular, the effects of mulching and basal N application depth on WUE, NUE and grain yield of maize on the Loess Plateau have not been investigated. The evaluation of N uptake and nitrate-N distribution under different basal N application depths is a new method to precisely assess N fertilizer management on farms. However, the interaction effects of mulching and basal N application depth on N uptake and nitrate-N distribution have never been reported. Therefore, an experiment was designed and conducted under field conditions. The aim of our investigation was to characterize the effects of plastic mulching and deep N fertilizer application on maize yield and N use efficiency, N uptake and nitrate-N distribution in the maize root layer.

## Materials and Methods

### Experiment Design and Management

A 2-year field study was carried out at the Gaoqiao Experimental Station (36°39′19.72″N, 109°11′26.26″E, altitude 1109 m) on the Loess Plateau in Ansai County of Shaanxi Province, China. The texture of the field soil was calcareous soil (USDA soil taxonomy). The average soil bulk density, organic matter, total carbon, total N, nitrate N, ammonium N, available phosphorus and available potassium were determined in April 2014 and April 2015 (**Table 1**). The average sand, silt and clay contents in the 0–30 cm soil depths were 23 ± 2.5, 63 ± 3, and 11 ± 1.5%, respectively; those in the 30–60 cm soil depths were 15 ± 1.5, 65 ± 2, and 18 ± 3%, respectively; and those in the 60–100 cm soil depths were 6 ± 1, 66 ± 3, and 25 ± 1.5%, respectively. The average annual precipitation in the study area for the 1952-2012 reference period was 531 mm, and the mean annual air temperature was 8.8°C. The data were collected at Yan’an experimental station meteorological observatory, and the weather station is located approximately 10 km from the experimental field. Precipitation and daily temperature for the growing season were recorded using an automatic weather station (HOBO Event Logger, United States).

**Table 1:** Major soil physicochemical characteristics of the experimental site measured before the experiment.

Year	Soil depth (cm)	Bulk density (g cm^-3^)	Organic matter (g kg^-1^)	Total C (g kg^-1^)	Total N (g kg^-1^)	NO_3-_-N (mg kg^-1^)	NH_4+_-N (mg kg^-1^)	Avail. P (mg kg^-1^)	Avail. K (mg kg^-1^)
2014	0–30	1.22	14.33	12.40	0.62	7.88	2.90	44.2	151.2
	30–60	1.28	9.67	11.60	0.44	3.89	2.40	26.1	137.1
	60–100	1.33	6.54	10.30	0.41	2.28	2.50	13.5	121.5
2015	0–30	1.20	12.63	11.10	0.71	9.83	2.80	38.7	144.6
	30–60	1.27	9.88	12.70	0.45	4.26	2.70	19.8	123.3
	60–100	1.32	5.49	10.00	0.39	3.18	2.00	16.6	114.5


The maize breed (*Zea mays* L., cv. ‘Shandan 609’) was cultivated in this experiment. This cultivar has been bred in China for maize grain production and selected for extensive planting and strong adaptation to climate and environmental conditions. The treatments were as follows: no N fertilizer and no mulching (CK), traditional broadcast basal N fertilizer with plastic mulching (T0), basal N-fertilizer application at a depth of 6 cm with no mulching (T1), basal N-fertilizer application at a depth of 6 cm with plastic mulching (T2), basal N-fertilizer application at a depth of 12 cm with no mulching (T3), and basal N-fertilizer application at a depth of 12 cm with plastic mulching (T4) (**Table 2**). Each treatment had three replications (18 plots in total). Each plot was 8 m long and 4 m wide, with an area approaching 600 m^2^.

**Table 2:** Treatment of mulching and basal fertilizer application method in this study.

Treatment	Mulching	Fertilizer rate (N-P-K kg ha^-1^)	Nitrogen application depth (cm)	Basal fertilizer application method
CK	No	0-80-80	–	First, spread the P and K fertilizer over the soil surface. Second, turn over the soil and mix the fertilizer into the soil.
T0	Yes	160-80-80	–	First, spread the basal N, P, and K fertilizer over the soil surface. Second, turn over the soil and mix the fertilizer into the soil. Third, cover the soil surface layer of the ridges (15 cm high × 60 cm wide) with plastic film mulch immediately after preparation.
T1	No	160-80-80	6	First, spread the P and K fertilizer over the soil surface. Second, turn over the soil and mix the fertilizer into the soil. Third, manually dig ditches to 6 cm depth. Fourth, perform band placement of basal N fertilizer and mark the fertilization position immediately after preparation.
T2	Yes	160-80-80	6	First, spread the P and K fertilizer over the soil surface. Second, turn over the soil and mix the fertilizer into the soil. Third, manually dig ditches to 6 cm depth. Fourth, perform band placement of basal N fertilizer. Fifth, cover the soil surface layer of the ridges (15 cm high × 60 cm wide) with plastic film mulch and mark the fertilization position immediately after preparation.
T3	No	160-80-80	12	First, spread the P and K fertilizer over the soil surface. Second, turn over the soil and mix the fertilizer into the soil. Third, manually dig ditches to 12 cm depth. Fourth, perform band placement of basal N fertilizer and mark the fertilization position immediately after preparation.
T4	Yes	160-80-80	12	First, spread the P and K fertilizer over the soil surface. Second, turn over the soil and mix the fertilizer into the soil. Third, manually dig ditches to 12 cm depth. Fourth, perform band placement of basal N fertilizer. Fifth, cover the soil surface layer of the ridges (15 cm high × 60 cm wide) with plastic film mulch and mark the fertilization position immediately after preparation.


Based on the local fertilizer application rates and considering prior research carried out in the region (Wang et al., 2015), all of the treatment plots received a basal fertilizer application of 80 kg P_2_O_5_ ha^-1^ and 80 kg K_2_O ha^-1^ in both years. The T0, T1, T2, T3, and T4 treatments entailed applications of 80 kg N ha^-1^ in both years. Urea (N, 46%), diammonium hydrogen phosphate (P_2_O_5_, 53%; N, 21%), and potassium sulfate (K_2_O, 52%; S_2_O, 40%) were used for fertilization. Plastic mulching was supplied by Yonggu Suye Co., Ltd., Shaanxi, China. For the treatments with plastic mulching, the soil surface layer of the ridges was laid immediately after preparation, with colorless and transparent 80-cm wide and 0.008-mm thick polyethylene film. The preparation and mulching were conducted approximately 1 week before maize sowing to minimize soil water loss for maize seed germination.

The maize seeds were manually sown on April 19th 2014 and 24th April 2015 and covered with a 2–3 cm soil layer. Maize was planted with 30-cm row and 40-cm line spacing. The basal N applications of different depths for the different treatments are presented in **Supplementary Figure S1**. In each plot, 8 rows of maize were hand sown to obtain a plant density of 60000 plants ha^-1^. This plant density is widely used in this region according to the recommendation of a local agricultural extension agency. Weeds, diseases and insect pests were rare at the field site; therefore, chemical control was not necessary. Top-dressing N (80 kg N ha^-1^) was manually applied in the middle of July to the treatments T0, T1, T2, T3, and T4. The maize crop was harvested on 28th September in 2014 and on 3rd October in 2015. The different growing seasons of maize are shown in **Supplementary Figure S2**. After harvest, the plastic film was gathered and recycled by the manufacturer. Subsequently, alfalfa was sown in all of the plots to balance the residual N-fertilizer among the different treatments.

### Grain Yield, Biomass, and Harvest Index

Grain yield and aboveground biomass were determined from a 4-m^2^ area in the middle rows of each plot at harvest time. Plants were weighted separately after dividing them into grains and stalks. All of the samples were oven-dried for 30 min at 105°C to quickly cease plant metabolic activities and then at 70°C to a constant weight to obtain the total aboveground biomass (kg ha^-1^). The harvest index was determined using the ratio of grain yield to total aboveground biomass yield.

### Soil Nitrate-N Content, Nitrogen Balance Estimation and Nitrogen Use Efficiency

Each year, soil nitrate-N content in the 100-cm profile at sowing and harvest was measured using a spectrophotometer (UV-VIS 8500, China) with three replications and with a sampling depth interval of 20 cm down to 100 cm. The potassium chloride extracting solution was 0.5 N, and the soil to solution ratio was 1:3. The methodology was according to Zavattaro L, Monaco S, Sacco D, and Grignani C (Zavattaro et al., 2012). The soil measurements were made in the horizontal direction at three observation points: 5 cm from the plants, toward the furrow at 20 cm and toward the ridge at 20 cm (**Supplementary Figure S3**). In this study, the soil nitrate-N below 100 cm soil depth and the ammonium-N throughout the whole soil profile was not included in the N content measurements because most of the crop roots were distributed within the 0–100 cm soil depth (Liu et al., 2003).

Total N content in grain and straw of the maize subsamples was determined by the micro-Kjeldahl method by digesting the sample in H_2_SO_4_–H_2_O_2_ solution (Barbano and Clark, 1990). N uptake by plants was estimated by multiplying grain and straw dry matter weight by their N concentrations (Fang et al., 2006).

The mass balance approach was used to assess the effect of biomass harvesting on N transport (Yang et al., 2016). N fertilizer application and soil N residual are the main sources of soil nitrate-N. The exported soil N is the N that has been lost from the system, which includes loss via plant N uptake and various N losses (Wu et al., 2013). Items in the N balance were estimated in each plot for the growing seasons from April to October for two consecutive years. For each period, the N balance was calculated as follows (Fang et al., 2006; Mekonnen et al., 2016):

(1) $$N_{initial}+N_{input}+N_{\min}=N_{uptake}+N_{residual}+N_{loss}(\mathrm{unit}:\;\mathrm{kg}\;{\mathrm{ha}}^{-1})$$

• *N_initial_* is the initial soil nitrate-N in the 0–100 cm soil profile (kg ha^-1^). The initial soil NO_3-_-N content is calculated as follows:

(2) $$N_{initial}=C_{1}\times h\times \rho \times 10\times 0.01$$

where *C*_1_ is the soil nitrate-N content (mg kg^-1^), *h* is the soil thickness (cm) and ρ is the soil bulk density (g cm^-3^).

• *N_input_* is the nitrogen fertilizer application level (0 or 160 kg N ha^-1^).
• *N_min_* is the nitrogen mineralization.
• *N_uptake_* is the nitrogen uptake by plants.
• *N_residual_* is the soil nitrate-N residual in the 0–100 cm soil profile.
• *N_loss_* is the soil nitrate-N loss in the 0–100 cm soil profile.

*N_loss_* mainly comes from soil nitrate-N leaching because other nitrogen losses via volatilization, denitrification, and erosion are low under environmental conditions similar to those of the present study (Sebilo et al., 2013; Chen et al., 2014; Windham-Myers et al., 2014).

Seasonal nitrogen mineralization (*N_min_*) was calculated as the balance of nitrogen fertilizer application level and output in the control treatment (CK, no N fertilizer and no mulching).

(3) $$N_{\min}=N_{uptake,0}+N_{residual,0}-N_{initial,0}(\mathrm{unit}:\;\mathrm{kg}\;{\mathrm{ha}}^{-1})$$

where *N*_*uptake*,0_, *N*_*residual*,0_, and *N*_*initial*,0_ are crop N uptake and residual and initial soil nitrate-N, respectively, in the 0–100 cm soil profile of the control treatment (CK).

Nitrogen recovery efficiency (*NRE*, in %) and nitrogen use efficiency (*NUE*, in kg kg^-1^) were analyzed using the following equation (Fiez et al., 1995; Šturm et al., 2010):

(4) $$NRE=\frac{N_{residual}-N_{initial}+N_{uptake}-N_{uptake,0}}{N_{input}}\times 100\%$$

(5) $$NUE=\frac{GY}{N_{input}}$$

where *GY* is the grain yield of maize (kg ha^-1^) and *N_input_* is the nitrogen fertilizer application rate (kg ha^-1^).

### Statistical Analysis

One-way ANOVA was used to assess the significance of differences among the means for each of grain yield, biomass accumulation, harvest index, N uptake by plants, soil nitrate-N residual, N loss, N recovery efficiency and N use efficiency in both years (followed by Tukey’s *b post hoc* test, significance level of 0.05). A general linear model (GLM) with plastic mulching (M), basal N application depth (D) and cropping year (Y) included as three fixed factors was used to assess variation in each of maize grain yield, biomass accumulation, harvest index, N uptake by plants, soil nitrate-N residual, N loss, N recovery efficiency and N use efficiency. The significance of difference between means was determined using the least significance difference (LSD) at *P* ≤ 0.05 in SPSS 16 (SPSS, Inc., United States).

### Weather Conditions

**FIGURE 1:**
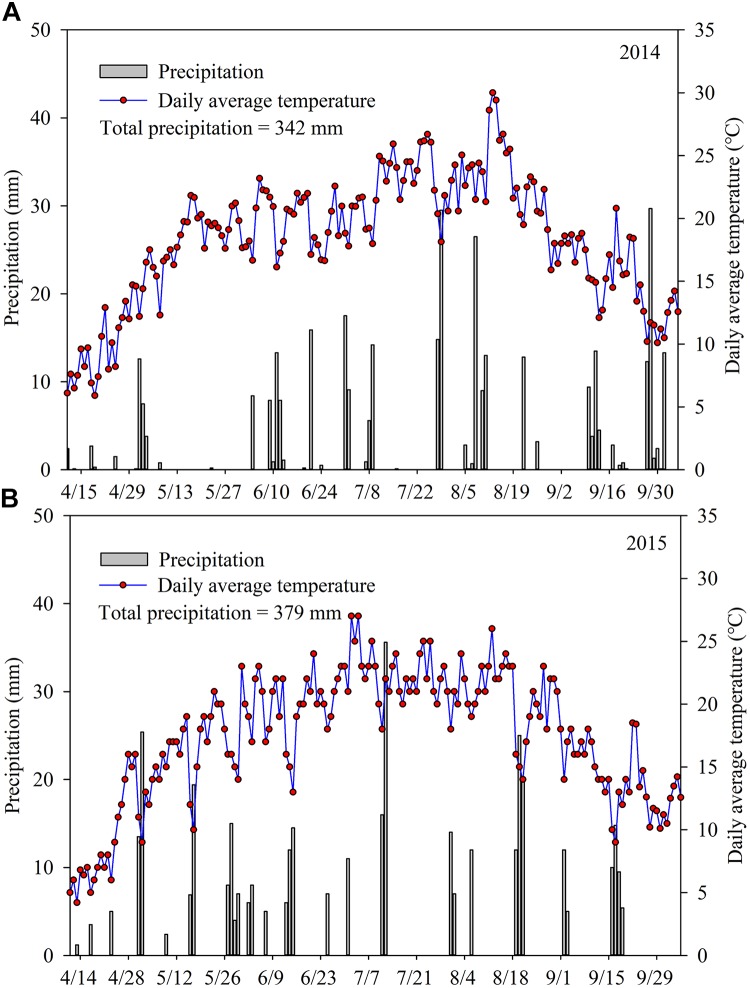
During the maize growing season, precipitation and daily average temperature were recorded in 2014 **(A)** and 2015 **(B)**.

The total rainfall in the maize growing season was 342 and 379 mm in 2014 and 2015, respectively (**Figure 1**). In June, more accumulated rainfall was observed in 2014 (76.2 mm) than in 2015 (56.7 mm). In 2015, the month with the greatest accumulated rainfall was May, with 78.1 mm, whereas only 44.9 mm was recorded in this month in 2014. In the maize growing season, the mean temperature was 18.4°C in 2014 and 17.7°C in 2015. The average daily temperature was above 20°C for 85 days in 2014 and 75 days in 2015, accounting for 52.5 and 46.3% of the whole maize growth period, respectively.

## Results

### Grain Yield, Biomass and Harvest Index

**FIGURE 2:**
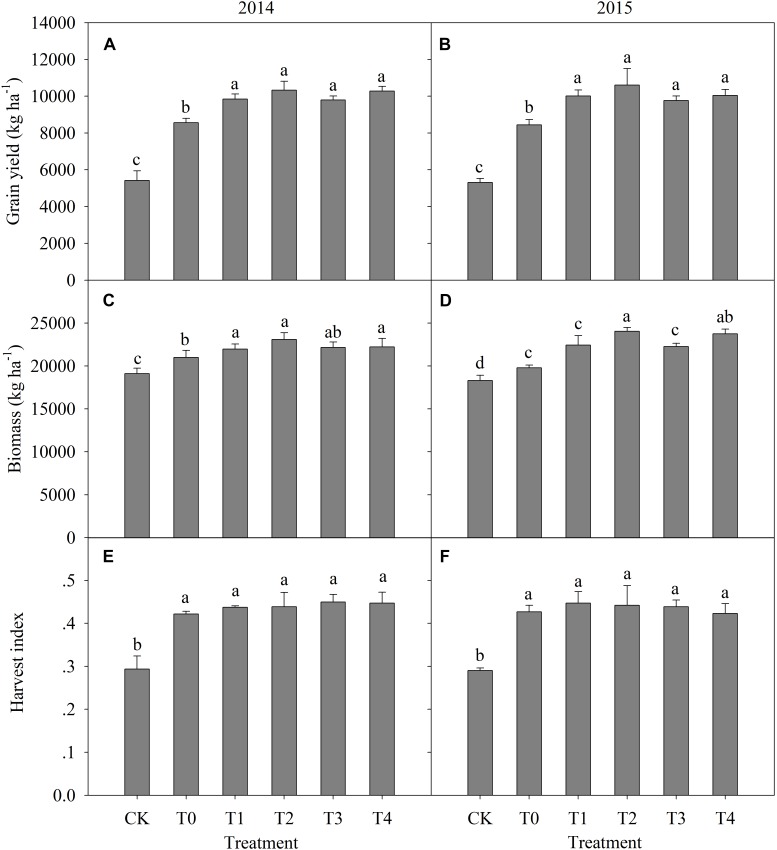
Effects of nitrogen rate, plastic mulching and basal nitrogen application depth on grain yield (**A**, 2014; **B**, 2015), biomass (**C**, 2014; **D**, 2015), and harvest index (**E**, 2014; **F**, 2015) of maize. Different letters in each section of a column indicate significant differences at *p* < 0.05. CK, no fertilizer and no mulching treatment; T0, traditional broadcast nitrogen fertilizer with mulching treatment; T1, basal nitrogen application at a depth of 6 cm with no mulching treatment; T2, basal nitrogen application at a depth of 6 cm with plastic mulching treatment; T3, basal nitrogen application at a depth of 12 cm with no mulching treatment; T4, basal nitrogen application at a depth of 12 cm with plastic mulching treatment.

The individual factors of mulching and N-fertilizer application depth significantly (*P* ≤ 0.01) affected grain yield and biomass accumulation, but there were no significant 2-way or 3-way interaction effects on grain yield or biomass accumulation (**Table 3**). In 2014, the highest grain yield was observed in T2 (10,336 kg ha^-1^), which was increased by 91% relative to the CK value (no N fertilizer and no mulching, 5425 kg ha^-1^) (**Figure 2A**). Plastic mulching and basal N application at the 12-cm depth (T4) produced a yield of 10,289 kg ha^-1^ in 2014, which was increased by 90% relative to the CK value (**Figure 2A**). Averaging across mulching and basal N-fertilizer application depths (from T1 to T4), the mean grain yield was increased by 15% in 2014 and 16.4% in 2015 relative to the grain yield in the T0 (traditional broadcast basal N-fertilizer with plastic mulching) treatment (**Figures 2A,B**). Among the mulching treatments, T0 resulted in a significantly lower grain yield than the yields obtained with basal N application at depths of 6 and 12 cm (T2 and T4). Grain yield in the T0 treatment was 15.1 and 14.4% lower than that in the T1 and T3 treatments, respectively, in 2014, and 18.6 and 15.6% lower than that in the T1 and T3 treatments, respectively, in 2015 (**Figures 2A,B**).

**Table 3:** Effects of plastic mulching (M), basal nitrogen application depth (D) cropping year (Y) and their interactions on maize grain yield (GY), biomass accumulation (BA), harvest index (HI), nitrogen uptake by plants (NUP), soil nitrate-N residual (NR), nitrogen loss (NL), nitrogen recovery efficiency (NRE) and nitrogen use efficiency (NUE).

Source	GY	BA	HI	NUP	NR	NL	NRE	NUE
Mulching (M)	0.01	<0.001	NS	0.013	<0.001	<0.001	<0.001	0.013
Depth (D)	<0.001	<0.001	NS	<0.001	<0.001	<0.001	<0.001	<0.001
Year (Y)	NS	NS	NS	NS	0.037	NS	NS	NS
M × D	NS	NS	NS	NS	NS	NS	NS	NS
D × Y	NS	NS	NS	NS	NS	NS	NS	NS
Y × M	NS	NS	NS	NS	NS	NS	NS	NS
M × D × Y	NS	NS	NS	NS	NS	NS	NS	NS


*NS, not significant (P > 0.05)*.

Mulching generally increased biomass accumulation, with the mean biomass in T2 and T4 being 5.9 and 6.2% higher than that in T1 and T3, respectively, over the 2 years (**Figures 2C,D**). In both years, the T2 treatment produced the maximum biomass accumulation, followed by the T4 treatment (**Figures 2C,D**). Averaging across years, the order of mean biomass accumulation from high to low was T2 > T4 > T1 > T3 > T0 > CK (**Figures 2C,D**). Averaging across mulching and basal N-fertilizer application depths (from T1 to T4), the mean biomass accumulation was increased by 18.6% in 2014 and 20.9% in 2015 relative to the corresponding CK value (**Figures 2C,D**). Thus, the harvest index in the CK treatment was only 0.29 in both years, which was significantly lower than the values in the other treatments (**Figures 2E,F**). Averaging across years, mulching and basal N-fertilizer application depths, the mean harvest index (from T1 to T4) was 34% higher than that in the CK treatment (**Figures 2E,F**).

### Soil Nitrate-N Content

**FIGURE 3:**
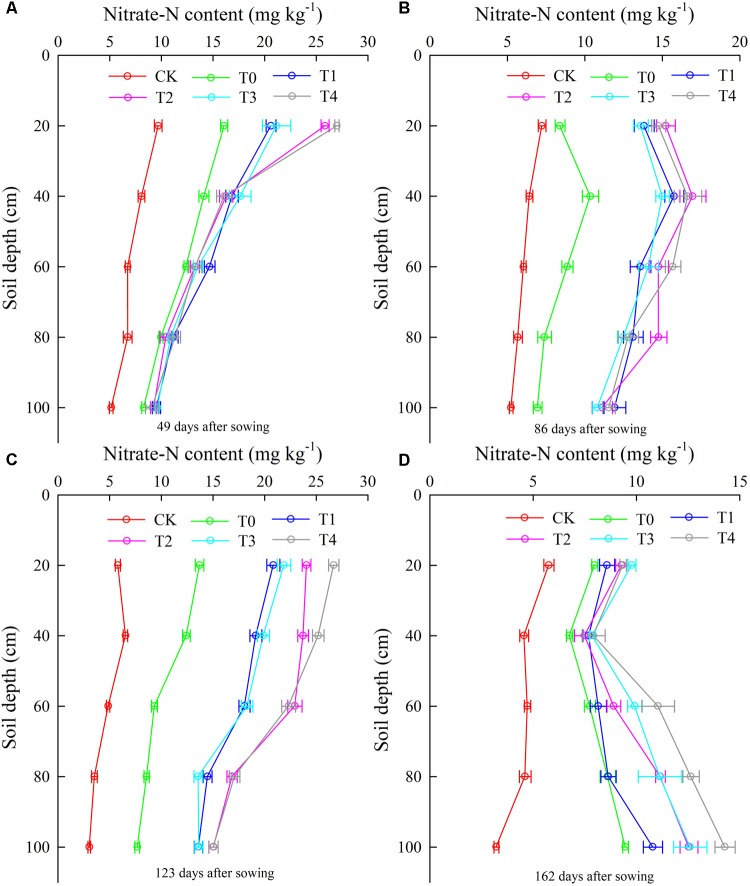
The vertical distribution of nitrate-N content was affected by plastic mulching and fertilization mode at different growth stages (**A**, 49 days after sowing, **B**, 86 days after sowing, **C**, 123 days after sowing, and **D**, 162 days after sowing) in 2014 (mg kg^-1^). CK: no fertilizer and no mulching treatment; T0, traditional broadcast nitrogen fertilizer with mulching treatment; T1, basal nitrogen application at a depth of 6 cm with no mulching treatment; T2, basal nitrogen application at a depth of 6 cm with plastic mulching treatment; T3, basal nitrogen application at a depth of 12 cm with no mulching treatment; T4, basal nitrogen application at a depth of 12 cm with plastic mulching treatment.

The nitrate-N content ranged from 5 to 25 mg kg^-1^, and the nitrate-N content decreased with increasing soil depth at 49 days after sowing in 2014 (**Figure 3A**). Averaging across basal N-fertilizer application depths and soil depths, the mean nitrate-N content for basal N-fertilizer application depths of 6 and 12 cm was 18.2% (2.7 mg kg^-1^) and 51.1% (7.6 mg kg^-1^) higher than those T0 and CK, respectively; at 49 days after sowing in 2014. Mulch generally increased the nitrate-N content; averaging soil depths, mulch increased the nitrate-N content by 3.2% in the treatment with an N-fertilizer application depth of 6 cm and by 5.1% in the treatment with an N-fertilizer application depth of 12 cm (**Figure 3A**). The nitrate-N content first increased and then decreased with increasing soil depth, and the highest mean nitrate-N content was observed at a depth of 60 cm during 86 days after sowing in 2014 (**Figure 3B**). Averaging across soil depths and mulching approaches, the basal N-fertilizer application depth of 6 cm increased the nitrate-N content by 40.1%, and that of 12 cm increased the content by 39% (**Figure 3B**). Mulching generally increased the nitrate-N content at 86 days after sowing in 2014 (**Figure 3B**).

**FIGURE 4:**
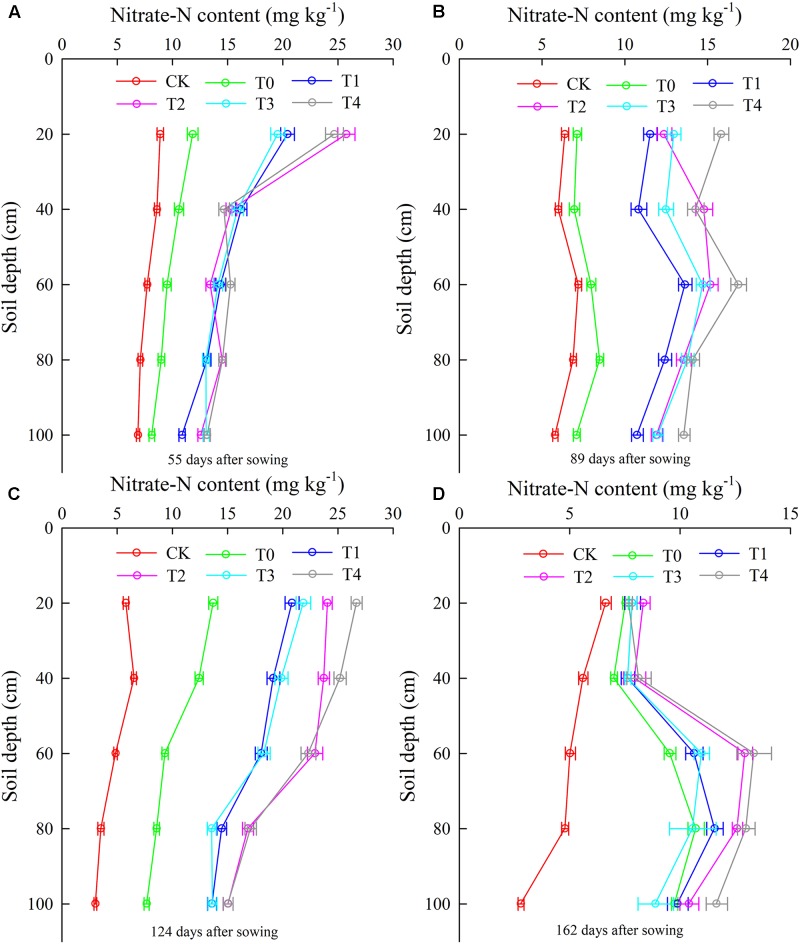
The vertical distribution of nitrate-N content was affected by plastic mulching and fertilization mode at different growth stages (**A**, 55 days after sowing, **B**, 89 days after sowing, **C**, 124 days after sowing, and **D**, 162 days after sowing) in 2015 (mg kg^-1^). CK, no fertilizer and no mulching treatment; T0, traditional broadcast nitrogen fertilizer with mulching treatment; T1, basal nitrogen application at a depth of 6 cm with no mulching treatment; T2, basal nitrogen application at a depth of 6 cm with plastic mulching treatment; T3, basal nitrogen application at a depth of 12 cm with no mulching treatment; T4, basal nitrogen application at a depth of 12 cm with plastic mulching treatment.

After the topdressing N-fertilizer, the mean nitrate-N content generally increased, ranging from 10 to 25 mg kg^-1^. Nitrate-N content decreased linearly with increasing soil depth at 123 days after sowing in 2014 (**Figure 3C**). Averaging across basal N-fertilizer application depths and soil depths, the mean nitrate-N content for basal N-fertilizer application depths of 6 and 12 cm was 46% (8.8 mg kg^-1^) and 75.2% (14.4 mg kg^-1^) higher than those of T0 and CK, respectively, at 123 days after sowing in 2014 (**Figure 3C**). Mulching greatly increased the nitrate-N content at 123 days after sowing in 2014, with increases of 19.2% for the basal N-fertilizer application depth of 6 cm and of 21.9% for that of 12 cm, averaging across soil depths (**Figure 3C**). Averaging across soil depths, the mean nitrate-N contents in T2 and T4 were 12.5 and 7.7% higher than those T1 and T3, respectively, at 162 days after sowing in 2014 (**Figure 3D**). A lower nitrate-N content was obtained near the root system of maize (20–40 cm), first decreased and then increased with increasing soil depth (**Figure 3D**). A high nitrate-N content was observed at 60 cm (**Figure 3D**). A similar result was obtained for 2015 (**Figure 4**), although at a soil depth of 40 cm, a lower nitrate-N content was observed in 2015 than in 2014 (**Figures 3B, 4B**).

### Nitrogen Balance Estimation and Nitrogen Use Efficiency

The highest N uptake was observed in the T2 treatment, which was 21.3 and 25.3% higher than that in the T0 treatment in 2014 and 2015, respectively (**Table 4**). Considering the basal N application depth, the N uptake of the T2 treatment was 7.3% higher than that of the T4 treatment in 2015 (**Table 4**). The N residual varied between 98 to 118 kg ha^-1^ in 2014 and between 108 to 128 kg ha^-1^ in 2015 (**Table 4**). The individual factors of mulching and N-fertilizer application depth significantly (*P* < 0.001) affected the N residual, and there was also a significant effect of year on the N residual (**Table 3**). The lowest N loss was observed in the treatment with plastic mulching and basal N-fertilizer application at the 6 cm depth in both years (**Table 4**). N loss in the T0 treatment was 34.6 and 39.8% higher than the corresponding values in the T1 and T3 treatments in 2014 (**Table 4**).

**Table 4:** Soil starting nitrogen (*N_initial_*), nitrogen input (*N_input_*), nitrogen mineralization (*N_min_*), nitrogen uptake by plants (*N_uptake_*), soil nitrate-N residual (*N_residual_*) in the 0–100-cm profile, nitrogen loss (*N_loss_*), nitrogen recovery efficiency (NRE) and nitrogen use efficiency (NUE) as affected by nitrogen rate, plastic mulching and basal nitrogen application depth in the maize growing season in 2014 and 2015.

Treatment		*N_initial_* (kg ha^-1^)	*N_input_* (kg ha^-1^)	*N_min_* (kg ha^-1^)	*N_uptake_* (kg ha^-1^)	*N_residual_* (kg ha^-1^)	*N_loss_* (kg ha^-1^)	NRE (%)	NUE (kg kg^-1^)
2014	CK	56	0	43	52 (0.8) d	47 (0.8) d	–	–	–
	T0	56	160	43	91 (5.5) c	98 (7.9) c	70 (5.3) a	51 (3.3) d	53 (1.5) b
	T1	56	160	43	109 (3.3) ab	105 (6) bc	46 (5) b	66 (3.1) c	62 (1.7) a
	T2	56	160	43	116 (8.6) a	117 (8.2) a	26 (16) d	78 (10.1) a	65 (3) a
	T3	56	160	43	105 (4) b	112 (4.6) ab	42 (5.3) bc	68 (3.3) bc	61 (1.4) a
	T4	56	160	43	115 (5) a	114 (5.2) ab	30 (4.5) cd	76 (2.8) ab	64 (1.5) a
2015	CK	68	0	49	55 (2.4) c	64 (2.4) c	–	–	–
	T0	68	160	49	87 (4.2) b	109 (5.5) b	81 (3) a	46 (1.9) c	53 (1.8) b
	T1	68	160	49	103 (7.3) a	108 (3.6) b	66 (10.6) b	55 (6.6) b	63 (2.1) a
	T2	68	160	49	117 (15.1) a	123 (6.5) a	37 (10.9) c	73 (6.8) a	66 (5.6) a
	T3	68	160	49	106 (8.1) a	108 (5.4) b	63 (6.2) b	57 (3.9) b	61 (1.6) a
	T4	68	160	49	108 (7.9) a	127 (7.1) a	42 (8.2) c	70 (5.2) a	63 (2) a


*Means within columns followed by the same lowercase letters are not significantly different (P < 0.05) according to Tukey’s b tests. The numbers in parentheses are one standard error of the mean (n = 3)*.

In the current study, mulching approach and basal N application depth each significantly affected nitrogen recovery efficiency (NRE) and nitrogen use efficiency (NUE), but there was no significant interaction effect of plastic mulching and basal N application depth on NRE or NUE (**Table 3**). The highest NRE values were observed in the T2 (78% in 2014, 73% in 2015) treatment, and the lowest values were observed in the T0 (51% in 2014, 46% in 2015) treatment (**Table 4**). Mulching greatly increased NRE and NUE (**Table 4**). Averaging across years, relative to the NRE value in the CK treatment, mulching increased NRE by 25.4% in the treatment with a basal N-fertilizer application depth of 6 cm and by 16.7% in that with an application depth of 12 cm. Furthermore, mulching increased NUE by 10% in the treatment with a basal N-fertilizer application depth of 6 cm and by 6% in that with an application depth of 12 cm (**Table 4**). The NUE in the treatment with plastic mulching and basal N application treatment at the 6 cm depth (T2) was 20.8 and 25.7% higher than that in the traditional broadcast N fertilizer (T0) treatment in 2014 and 2015, respectively (**Table 4**). With basal N application treatment at the 12 cm depth, NUE with plastic mulching was 5.1% higher than that in the no-mulching treatment in 2014 (**Table 4**). However, plastic mulching did not significantly increase NUE relative to the CK value in the treatment with basal N application at the 12 cm depth in 2015 (**Table 4**).

## Discussion

### Effects of Plastic Mulching and Basal Nitrogen Application Depth on Grain Yield, Biomass and Harvest Index

The results revealed that basal N-fertilizer application at depths of 6 and 12 cm enhanced grain yield and biomass in maize. Grain yield was significantly affected by the depth of basal N-fertilizer application. Generally, there is a positive correlation between grain yield and N application rate. However, there are few reports of the effect of basal N-fertilizer application depth on grain yield in maize. In this study, the grain yields in treatments with basal N-fertilizer application depths of 6 cm (T1, T2) and 12 cm (T3, T4) were significantly higher than the grain yield under treatment with traditional broadcast N fertilizer (T0) (**Figure 2**). This result demonstrates that the basal N application depth influenced maize yield. It is possible to reduce the amount of N released into the environment by NH_3_ volatilization and denitrification when N-fertilizer is applied to the subsurface (Guo et al., 2008a). One reason for the significant effect of N application depth on maize yield might be related to the lower nitrate-N content in the root absorption area than in other areas (**Figures 3, 4**), which reflects the fact that large amounts of N are absorbed during the maize grain filling stage (Guo et al., 2008b; Wang and Xing, 2016). We speculate that basal N-fertilizer application at depths of 6 and 12 cm provides sufficient soil nitrate-N for maize growth before the grain filling stage and that this uptake is conducive to the translocation of N from the vegetative organs to the grain during the filling stage.

In this study, the grain yield under N-fertilizer application at the depth of 6 cm was slightly higher than that at the 12 cm depth. There are two possible explanations for this phenomenon. One potential reason is that deep application of N fertilizer may increase the risk of N leaching (Dolan et al., 2006). Alternatively, a deeper N application depth may produce a higher N residual (**Table 4**). The soil water content significantly affects nitrate-N leaching, and a 5-year experiment proved that optimal N fertilization management contributes to high crop yields (Yang et al., 2015).

Data obtained from this 2-year study showed that plastic mulching treatment increased grain yield over that of no-mulching treatment at the same N-fertilizer application depth, and the added value of mulching in improving grain yield by mulching decreased with increasing basal N-fertilizer application depth (**Figure 2**). Plastic mulching increases maize grain yield by increasing soil water, which can stimulate maize root growth and promote a higher use efficiency of soil nitrate-N (Sharma et al., 2011; Rhodes, 2014). Higher yields were observed in the treatments with plastic mulching and N-fertilizer application, and the basal N-fertilizer application depth significantly affected grain yield. We conclude that plastic mulching can significantly increase maize production when basal N-fertilizer is applied at depths of 6 and 12 cm. There were no significant interaction effects of plastic mulching and basal N-fertilizer application depth on grain yield.

Only one maize breed was cultivated in this experiment; the results show that basal N-fertilizer application depths of 6 and 12 cm increased grain yield in this maize breed. It is important to conduct future studies on the effect of basal N-fertilizer application depth on different maize breeds.

### Effects of Plastic Mulching and Basal Nitrogen Application Depth on Nitrogen Use

Plastic mulching increased N uptake in this study. The treatments with basal N-fertilizer application at 6 (T1, T2) and 12 cm depths (T3, T4) yielded significantly higher N uptake than did broadcast basal N-fertilizer application (T0) in both years (**Table 4**). In addition, the results indicated that treatment with plastic mulching and basal N application at 6 cm or 12 cm depth significantly reduced the N loss (**Table 4**). One possible explanation for the higher N uptake under subsurface basal N-fertilizer application (T1 to T4) is that the deep placement of N fertilizer greatly reduces ammonia volatilization and denitrification loss (Cai et al., 2002). Another reason may be that the nitrate-N concentrations at the root zone were higher in the deep-placement treatments (band placement of basal N-fertilizer) than in the traditional broadcast basal N-fertilizer application treatment (Liu et al., 2003). Root growth in maize is better in soils of high nitrate-N content than those of low nitrate-N content and is associated with greater numbers and branching of root hairs (Fageria and Baligar, 2005).

When evaluating the effects of N-fertilizer management in the field (Ding et al., 2015), NUE is an important indicator, with high NUE being a criterion for sustainable agriculture (Liu et al., 2016). In the present study, NUE was significantly higher in the plastic mulching treatment than in the no-mulching treatment at the same basal N-fertilizer application depth, and the highest NUE was observed in the treatment with plastic mulching and basal N application at the 6 cm depth (T2) (**Table 4**). NUE in the deep-placement treatments (T1 to T4) was significantly higher than that in the surface broadcast treatment (T0). It has been reported that increasing NUE can reduce the risk of nitrate leaching (Li et al., 2007), as in general, less than half of applied N-fertilizer is absorbed by the plant (Franzen et al., 2016). The remainder N fertilizer is subjected to loss (Zhao et al., 2013), whereas a small part of the N fertilizer remains in the upper 60 cm of the soil profile (Wienhold et al., 1995). Our data clearly show that the NUE values were higher and the N uptake rate and N loss were lower in the deep-placement treatments (T1 to T4) than in the surface broadcast treatment (T0). This clearly shows that basal N-fertilizer application in the deep soil layers leads to a marked increase in NUE. However, this type of fertilizer management would require much more labor in the absence of the implementation of mechanization than does traditional management. Therefore, we recommended a basal N-fertilizer application depth of 6 cm for maize crops. The effects of basal N-fertilizer application depth at 6 cm in different maize breeds warrant further study.

## Conclusion

Averaging over mulching approaches and basal N-fertilizer application depths, mean grain yield under basal N-fertilizer application (T1, T2, T3, and T4) was 15–16.4% higher than that under traditional broadcast N fertilizer (T0) in 2014 and 2015, respectively. Plastic mulching produced a higher nitrate-N content than did no mulching in both years. N uptake from the plastic mulching treatment with basal N application at the 6 cm depth (T2) was 21.3 and 25.3% higher than that under traditional broadcast N fertilizer treatment (T0) in 2014 and 2015, respectively. Treatment with plastic mulching and basal N application at 6 cm or 12 cm depth significantly reduced N loss. Regardless of the research achievements and promotional activities of field management, traditional broadcast N fertilizer remains common practice, and considerable efforts will be needed to achieve widespread usage of basal N application in deep layers. Basal N application at a depth of 6 cm with plastic mulching treatment, as described here, is recommended because it significantly increased grain yield and NUE over those achieved under traditional fertilization in the present study.

## Author Contributions

XW conceived and designed the study and wrote the manuscript. XW and YX collected and analyzed the data. All of the authors discussed the results and commented on the contents of the manuscript.

## Conflict of Interest Statement

The authors declare that the research was conducted in the absence of any commercial or financial relationships that could be construed as a potential conflict of interest.
