# Supplementary material for: Effects of Plastic Mulching and Basal Nitrogen Application Depth on Nitrogen Use Efficiency and Yield in Maize
Source: Front Plant Sci. 2018 Oct 2;9:1446. doi: 10.3389/fpls.2018.01446 (PMC6176046; doi:10.3389/fpls.2018.01446)

Effects of plastic mulching and basal nitrogen application depth on nitrogen use efficiency and maize yield

Xiukang Wang*, Ning Wang, Yingying Xing, Jia Yun, Huihui Zhang

College of Life Sciences, Yan'an University, Yan'an, Shaanxi 716000, China

*Corresponding authors: wangxiukang@126.com

Address: 580 Shengdi Road, Yan'an, Shaanxi 716000, China

Tel/Fax: +86 911 2332030

Email: wangxiukang@126.com

Fig. 1S A sketch of the width direction arrangement and depth of basal nitrogen application in different treatments.

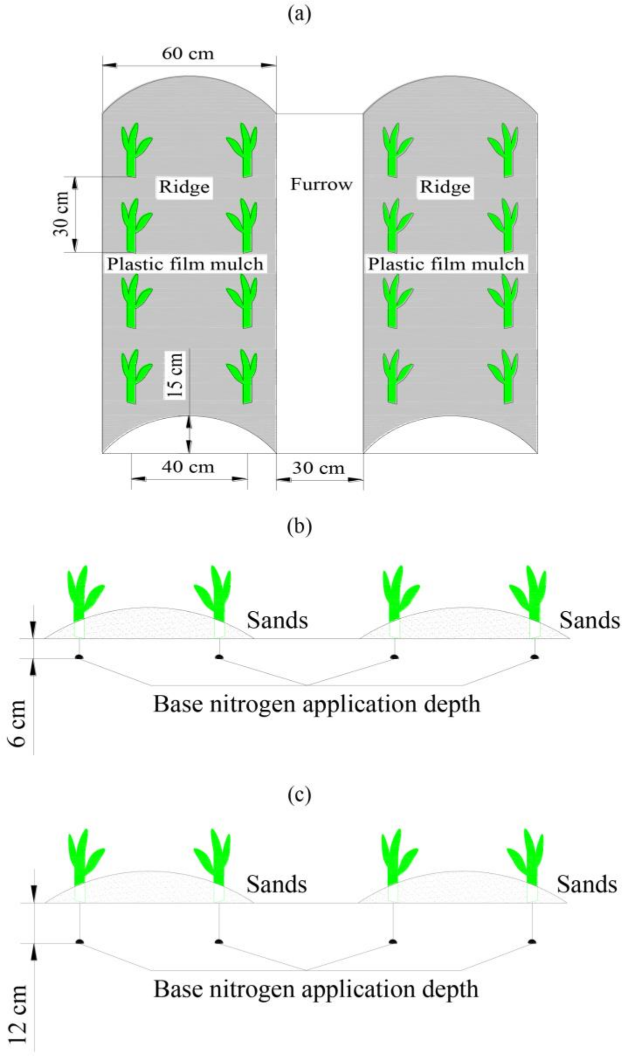

Fig. 2S Pre-planting, seasonal schedule of crop growth stage and harvest in the experiment.

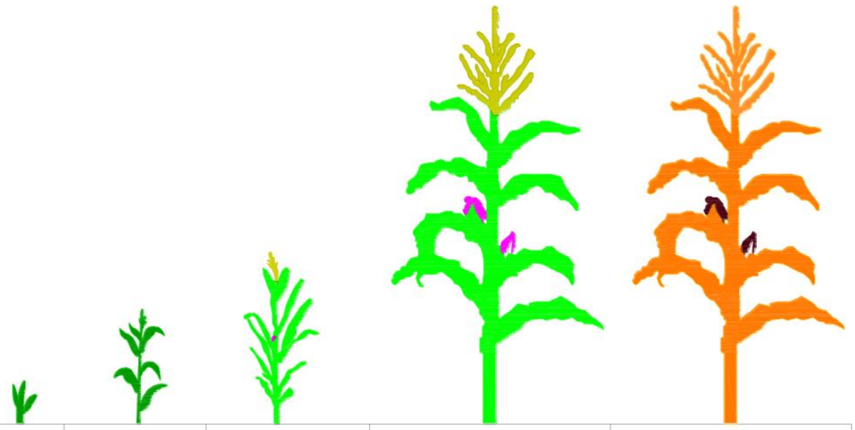

Pre-planting	Sowing	Jointing stage	Flowering stage	Filling stage	Maturity stage	Harvested
2014	Apr 19	Jun 07	Jul 14	Aug 20	Sep 28	
2015	Apr 24	Jun 18	Jul 22	Aug 26	Oct 03	

Fig. 3S Diagrammatic sketch of soil sampling locations in the soil profile for crop root zone.

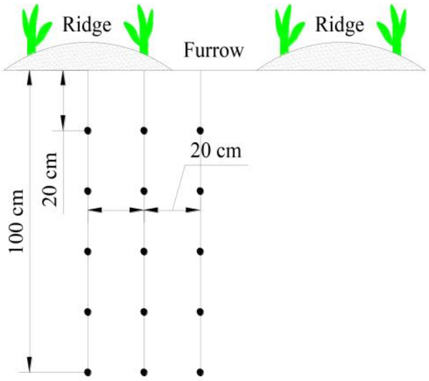

Supplement: Supplementary file 1 [file Data_Sheet_1.PDF]
